# Infection rates, risk factors and microbial etiology of Cerebrospinal Fluid (CSF) shunt infections – a single centre prospective study from India

**DOI:** 10.1017/ash.2025.146

**Published:** 2025-09-03

**Authors:** Dinoop Korol Patambah

## Abstract

**Background/Objectives:** CSF shunts are widely used in neurosurgery practice for temporary or permanent CSF diversion. Patients on CSF shunts are at risk of device-associated CNS infections particularly ventriculitis or meningitis. The study objectives were to delineate the risk factors and infection rates for various shunt procedures and their microbial etiology. **Methods:** This is a single center prospective cohort study. The study period was 2 years (October 2020- September 2022). Patients were categorised using IDSA criteria as Contamination or Colonisation or Infection. Device days were also collected from the Hospital information system (HIS) for calculation of infection rates. Microbial etiology was identified by culture of CSF and shunt catheter tips. Cox regression model was used to estimate hazard risk for various risk factors. **Results:** During the 2-year study period, 161 shunts were inserted.133 were ventriculo-peritoneal (VP) shunts, 19 were lumbo-peritoneal (LP) shunts, 6 were subduro-peritoneal (SDP) shunts, 2 were syringo- subarachnoid (SS) shunt and 1 cysto-peritoneal (CP) shunt. Hydrocephalus was the commonest indication for a shunt insertion (71.4 %). There were 8 VP shunt and 1 LP shunt infections during the study period. The average infection rates for VP and LP shunts were 6 and 5.2 per procedure, respectively. Gram negative bacteria caused most of the shunt infections (7/9, 77%). The most common organism causing shunt infection was *Klebsiella pneumoniae* (n=4, 44%), followed by *Staphylococcus aureus* (n=2, 22%). The risk factors which were independently associated with increased risk for shunt infection were Pre-OP ASA score >3 [HR:8.28, p - 0.013], presence of associated perioperative systemic [HR:3.89, p-0.01] or scalp infections [HR:3.53, p-0.005]. **Conclusion:** VP and LP shunt infection rates were similar in our study. *Klebsiella pneumoniae* was the commonest causative agent causing shunt infection. High Pre-OP ASA score and associated perioperative scalp or systemic infections were independent risk factors for shunt infection.

